# Super Ultra-High Resolution Liquid-Crystal-Display Using Perovskite Quantum-Dot Functional Color-Filters

**DOI:** 10.1038/s41598-018-30742-w

**Published:** 2018-08-27

**Authors:** Yun-Hyuk Ko, Mohammed Jalalah, Seung-Jae Lee, Jea-Gun Park

**Affiliations:** Advanced Semiconductor Materials and Devices Center, Department of Electronics and Computer Engineering, Hanyang University, Seoul, 133-791 Republic of Korea

## Abstract

Quantum dot enhancement film (QDEF) working in tandem with a blue light-emitting-diode (LED) back-light-unit (BLU) has been recently used in liquid crystal display (LCD) to minimize the cross talks between the polarized emitting B-, G-, and R-light. However, they still exhibit a fundamental and considerable emitting-light-power loss from QDEF because of the light absorption loss in resin and transparent films of QDEF. In this work, we propose and demonstrate the superiority of the LCD using blue-(B-), green-(G-), and red-(R-) perovskite-quantum-dot (PrQD) functional CFs coupled with a blue LED BLU. This LCD using PrQD functional CFs and a blue LED BLU features cross-talk free spectra of polarized emitting B-, G-, and R-light, maximizing the LCD color gamut and exhibiting a world record performance of over 102.7% (137%) of Rec.2020 standard (NTSC standard). Theoretically, such an improvement in color gamut would facilitate unlimited scaling-down of the pixel leading to super ultra-high resolution LCD.

## Introduction

**Figure 1:**
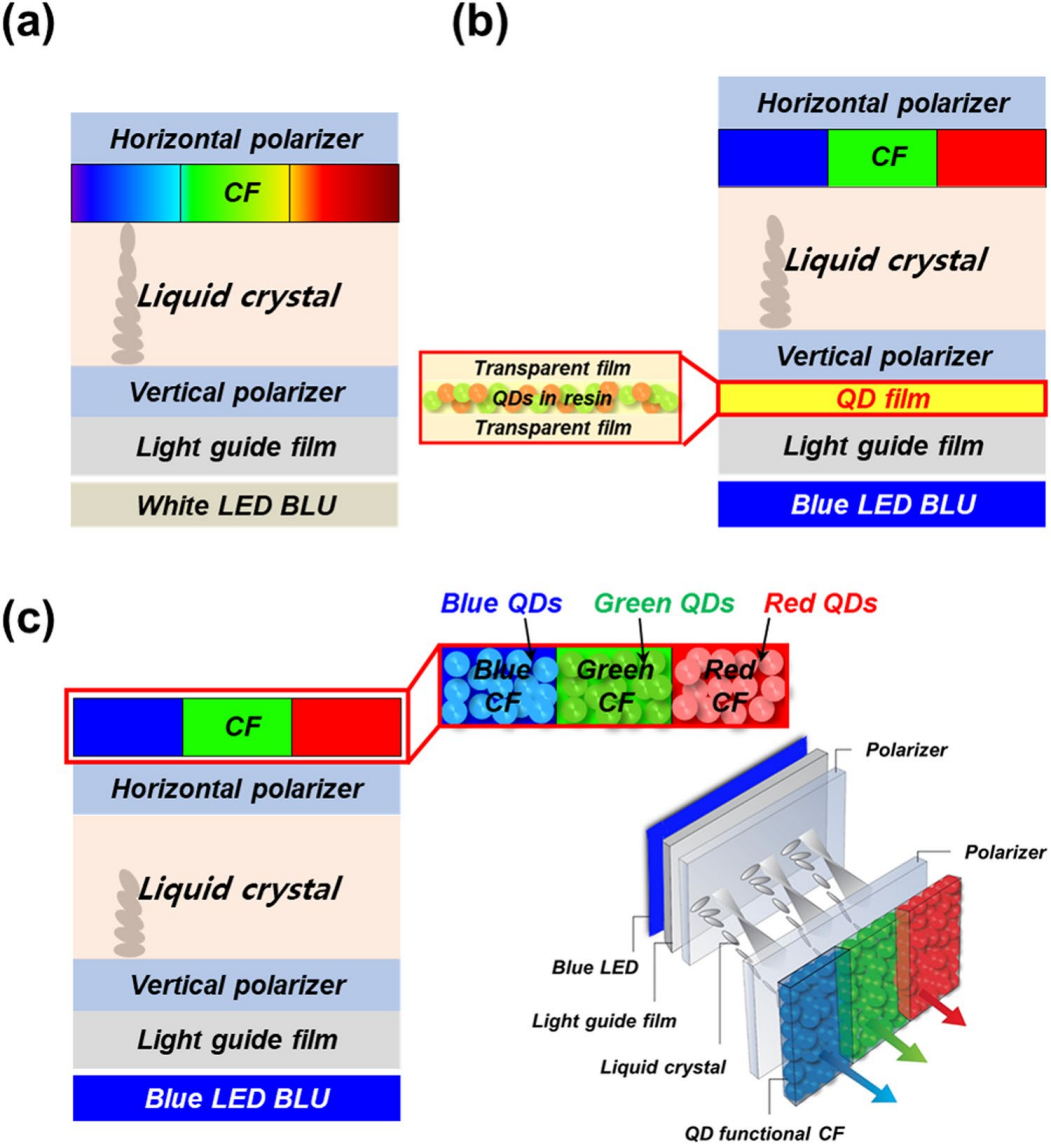
Schematic structures of liquid crystal displays (LCDs). (**a**) Traditional white LED backlight unit, (**b**) Embedded with a quantum-dot enhancement film (QDEF) and white LED backlight unit, and (**c**), with quantum-dot functional color filter (QD-functional CF) and blue LED BLU. Zoomed image in (**b**,**c**) show the structure of QDEF and QD-functional CF, respectively. In addition, right image in (**c**) shows the 3D schematic structure of QD-functional CF LCD.

Since 1980’s, LCD has been utilized as a main display monitor for desktop computer, note, and mobile phone etc. In the LCD technology, there was the first paradigm shift, called white light-emitting-diode (white LED) LCD, by changing from the BLU using white lamps to the BLU using white LEDs to achieve the advantages of low cost, high efficiency, long-term lifetime, high stability, and simple optical configuration^1–3^, as shown in Fig. 1a. Recently, the second paradigm shift in the LCD technology has introduced enthusiastically via using a new BLU technology, called quantum dot enhancement film (QDEF) LCD. This new BLU technology is fabricated with a tandem structure of a transparent film, transparent resin embedded with green and red-light-emitting quantum-dots (G and R-QDs), transparent film, and the blue LED illumination (~450 nm). In particular, this technology can improve the broad emitting blue (B), green (G), and red (R)-light spectrum of a white LED LCD limiting color gamut and color resolution, as shown in Fig. 1b^4–6^. Although this QDEFLCD technology can display almost cross-talk free emissions between B-, G-, and R-light emission, it has fundamentally a quite high emitting-light power loss (>10%) from the QDEF absorbing the blue LED illumination due to the light absorption loss in resin and transparent films of QDEF, as shown in Supplementary Fig. S1 (Supproting information). To overcome the fundamental drawback of QDEF LCD, herein, the third paradigm shift in the LCD technology is proposed by using B-, G-, and R-QD-functional CFs and blue LED BLU, called QD functional CF LCD, as shown in Fig. 1c. In addition, B-, G-, and R-pixels in the LCD using B-, G-, and R-QD-functional CFs can be patterned by using a conventional CF fabrication process. In particular, we tested the superiority of the LCD performance for our proposed QD-functional CF LCD in Fig. 1c, where blue LED BLU, light guide film, vertical polarizer, twisted nematic liquid- crystals, horizontal polarizer, and QD functional CF were vertically stacked and the LCD performance was estimated by polarizer microscope. In our research, B-, G-, and R-QD-functional CFs were fabricated by using core-structure perovskite-based QDs (PrQDs), showing a high photoluminescence-quantum yield (PL-QY) and a narrow full-width at half-maximum (FWHM) as well as a larger flexibility in tuning the suitable emitting PL peak wavelength from QDs with the transmitted light peak wavelength from CFs^7–13^. Under the expose of blue LED BLU, the LCD optical and color-gamut performance of B-, G-, and R-PrQD functional CFs was compared with those of conventional B-, G-, and R-CFs and B-, G-, and R-PrQDEF LCD.

## Materials and Methods/Experimental Procedures

### Materials and Chemicals

Oleic acid (OA, 90%), oleylamine (OLA, 70%), 1-octadecene (ODE, 90%), cesium carbonate (Cs_2_CO_3_, 99.9%), lead(II) chloride (PbCl_2_, 98%), lead(II) bromide (PbBr_2_, 98%), lead(II) iodide (PbI_2_, 99.999%), tert-butanol(tBuOH, 99.0%), and hexane (99.0%) were used as purchased from Aldrich. Red, green, and blue color filters were used as purchased from DONGJIN SEMICHEM Co. Blue LEDs (Dongbu LED, Inc.) were used as excitation sources.

### Synthesis and Purification of CsPbX_3_ QDs

The CsPbX_3_ QDs were synthesized by adapting the synthesis process developed by Protesescu *et al*.^14^ 0.814 g of Cs_2_CO_3_ was loaded into 100 mL 3-neck flask along with 40 mL of ODE (1-octadecene) and 2.5 mL of oleic acid (OA), dried for 1 h at 120 °C, and then heated under N_2_ to 150 °C until all Cs_2_CO_3_ reacted with OA. Since Cs-oleate precipitates out of ODE at room temperature, it has to be preheated to 150 °C before injection. 5 mL of ODE and 0.188 mmol of PbX_2_ such as blue perovskite QD (PbCI_2_: PbBr_2_ = 0.094 mmol: 0.094 mmol), green perovskite QD (PbBr_2_: PbCl_2_ = 0.031 mmol: 0.157 mmol), red perovskite QD (PbBr_2_: PbI_2_ = 0.063 mmol: 0.125 mmol) or their mixtures were loaded into 100 mL 3-neck flask and dried under vacuum for 1 h at 120 °C. 0.5 mL of dried OLA and 0.5 mL of dried OA injected at 120 °C under N_2_. After complete solubilisation of a PbX_2_ salt, the temperature raised to 165 °C and 0.4 mL of Cs-oleate stock solution quickly injected and, 30 sec. later, the reaction mixture cooled by the ice-water bath. The crude solution cooled down with water bath and aggregated QDs separated by centrifuging. For B-, G-, and R- perovskite QDs, centrifugation at 0 °C or addition of tert-butanol (tBuOH) to the crude solution (ODE:tBuOH = 1:1 by volume) were found to be helpful for a complete precipitation. After centrifugation, the supernatant discarded and the particles dispersed in hexane forming long-term stable colloidal solutions. The schematic diagram of this synthesis process is shown in. Supplementary Figs S2–S4 (Supporting information).

### Characterization

The detailed morphology, size distribution, absorption, composition ratio, and PL for QDs were characterized by a transmission electron microscope (TEM) with an acceleration voltage of 200 kV, UV visible spectroscopy, X-ray diffraction (XRD), energy-dispersive x-ray spectroscopy (EDX) line-scan profile, and photo-luminescence spectroscopy using a HeCd laser source with a 325 nm wavelength. FWHM and absolute PL quantum yield were measured by QE-2100 (Otsuka electronics), and those were cross-checked with photo-luminescence spectroscopy using organic dyes (Rhodamine 6 G, QY = 95% in ethanol). For quantum yield measurements in photo-luminescence spectroscopy, optical density (i.e. 0.05 absorbance) was adjusted to 0.005 wt% in excitation of 325 nm. In addition, the quantum yield for QDs were confirmed by measuring time-resolve PL decay curves (Hamamatsu C11347-11 using 365-nm LED source and 1 MHz).

### QD functional CF fabrication and LCD Performance

To fabricate the QD functional CF, all PrQDs QDs were dissolved in solvent (i.e., hexane) at 20 wt %, and 0.3 mL of R, G, and B color filters (DONGJIN SEMICHEM Co. model number: DCR-TR711R, DCR-TR711G, and DCR-TR711B) were mixed with 0.3 mL of the as-prepared QD solutions in a 5 mL glass vial. A QDEF was fabricated by mixing G- and R-PrQDs with a Si resin where the QDs were dispersed in hexane solvent. The quartz glass substrates were cleaned by sonication in isopropanol and acetone (1:1) for 10 min and dried under N_2_ gas. After 1 cm × 1 cm guide-rings were attached to the quartz glass substrates, the prepared R-, G-, and B-CFs as a reference (called conventional R-, G-, and B-CFs), QDEF including G- and R-QDs, and the R-, G-, and B-color filters mixed with R-, G-, and B-QD solution (called QD functional CFs) were uniformly coated in a 1 cm^2^ quartz substrate. The conventional R-, G-, and B- CFs and the R-, G-, and B-QD functional CFs were dried at room temperature to harden the film on the quart glass. The conventional CF, QDEF, and QD functional CFs were implemented on a polarizer microscope (Nikon Inc.) like in Fig. 1(a–c) where a blue LED was then applied a BLU. Thus, the LCD has a vertically stacked structure of blue LED, light guide film, vertical polarizer, twisted nematic liquid-crystals, horizontal polarizer, and conventional or QD functional CF. Then, we estimated the QY and FWHM of the polarized emitting B-, G-, and R-PL spectrums using a polarizer microscope.

## Results and Discussion

**Figure 2:**
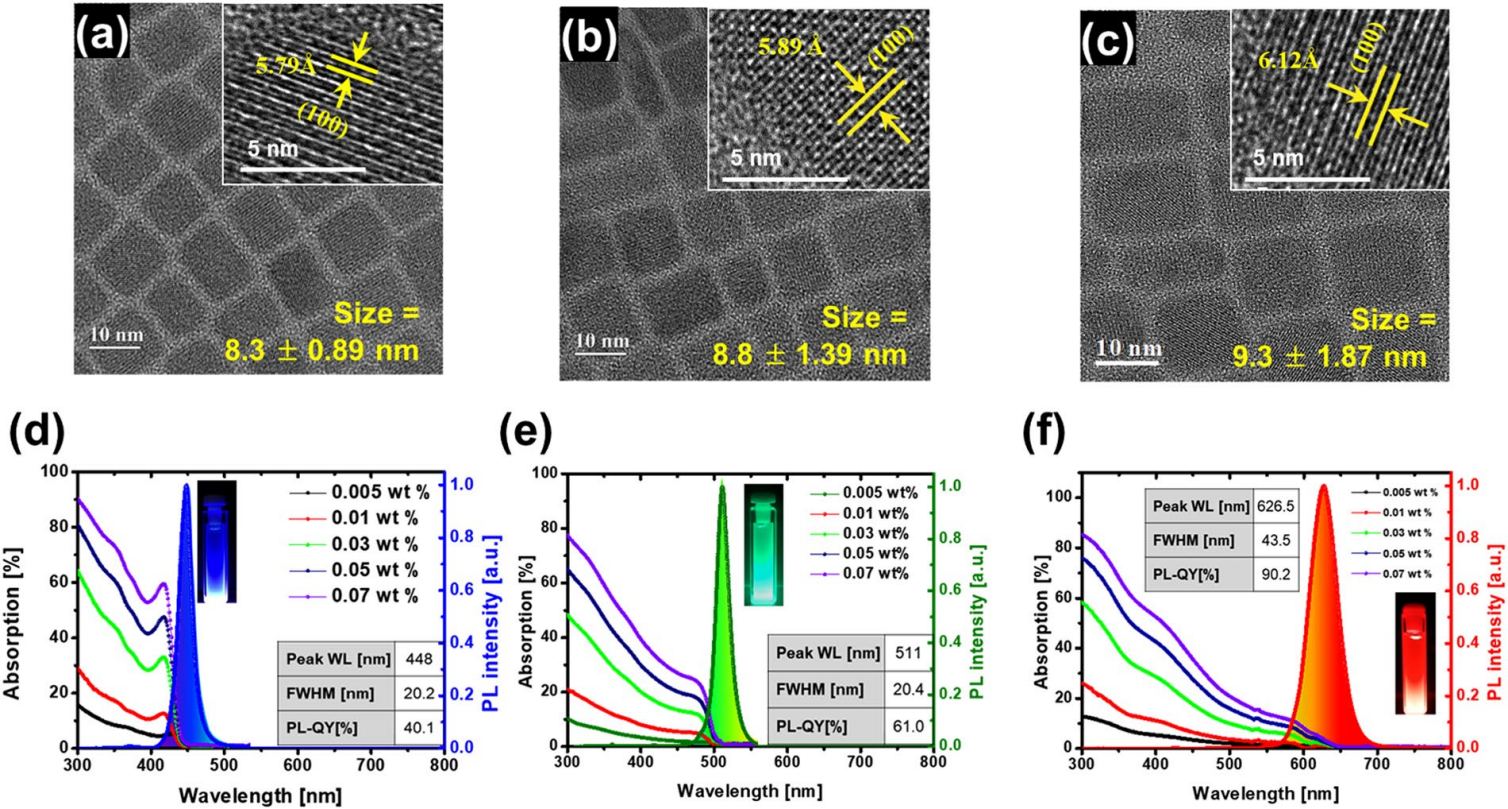
Morphology and optical properties of core structure perovskite-based QDs. HR-TEM images with a scale bar of 10 nm of B-PrQDs (**a**), G-PrQDs (**b**), and R-PrQDs (**c**). Inset images show the high-resolution TEM (HR-TEM) with a scale bare of 5 nm. Inset schematic image in (**d**–**f**) shows the cubic perovskite crystalline structure. Absorption and PL (at 0.005 wt%) B-PrQDs (**d**), G-PrQDs (**e**), and R-PrQDs (**f**) excited under 325 nm. Photographs (inset in **d**–**f**) of solution-based QDs under 365-nm hand-UV lamp for B-PrQDs, G-PrQDs, and R-PrQDs, respectively.

Three types of perovskite-based QDs, i.e. CsPbCl_1.4_Br_1.6_ (B-PrQD), CsPbCl_0.5_Br_2.5_ (G-PrQD), and CsPbBr_0.9_I_2.1_ (R-PrQD), were synthesized for QD-functional CFs to achieve narrow FWHM, especially, for blue and green light emissions in CF film state. These PrQDs were designed by adjusting the stoichiometric molar fraction between Cl-, Br- and I- anions to absorb the blue light of a blue LED and then emit only B-, G-, and R-light via energy-down-shift^15^. B-, G-, and R-PrQDs showed a cubical-like shape, high crystallinity, and well dispersity, as shown in HR-TEM images of Fig. 2a–c, indicating that the shape of PrQDs (i.e., cubical-like) is different from that of Cd and InP-based core/shell QDs (i.e., spherical-like)^13,15–17^ that are currently and commonly used in QDEF LCD. In addition, the diameters of B-, G-, and R-PrQDs were 8.3 ± 0.9, 8.8 ± 1.4, and 9.3 ± 1.9 nm, respectively. The lattice constant of B-, G-, and R-PrQDs were 5.8, 5.9, and 6.1 Å on the {100} plane, respectively, as shown in HR-TEM images of the insets in Fig. 2(a–c). It is noticed that the B-PrQD presented the smallest lattice constant while R-PrQD showed the highest value, which could be explained by the lower anionic radius of Cl^-^ presented in a high fraction in B-PrQD and the larger anionic radius of I^-^ existed in a high ratio in R-PrQD^18^. PrQDs have a cubic crystalline structure (JCPDS No. 54-0752)^19^, which was confirmed by SAED and XRD patterns, see Supplementary Fig. S5a–d (Supporting information). Note that the distinct rings originated from {100}, {110}, {200}, {210}, {220} in SAED were well consistent with the dominant XRD diffraction peaks for all three PrQDs. In addition, it is obvious that the positions of XRD diffraction peaks are influenced by the anions radii (i.e., a lower anion radius leads to a higher-2θ shift in XRD). For example, while the (110) peak was centered at 2θ of 31° for CsPbCl_1.4_Br_1.6_ (B-PrQD), it was detected at 29.4° for CsPbBr_0.9_I_2.1_ QD (R-PrQD), as shown in Supplementary Fig. S5d (Supporting information). The optical band gap of B-, G-, and R-PrQDs were 2.66, 2.38 and 1.92 eV, calculated by using Tauc plot, as shown in Supplementary Fig. S6 (Supporting information). Furthermore, the B-, G, and R-PrQDs absorbed the light less than 450, 550, and 650-nm-wavelength, respectively, and their absorbance in the solvent solution (i.e., hexane) increased with their concentration (wt%), measured by UV-visible spectroscopy, as shown in Fig. 2d–e. The B-, G, and R-PrQDs emitted the blue PL at 448, 511, and 627-nm-wavelength with a PL FWHM of 20.2 nm, 20.4 nm, and 43.5 nm and a PL-QY of 40.1%, 61.0%, and 90.2%, respectively. Note that the PL-QY of perovskite QDs could be further enhanced by applying silica or ZnS shell passivation^20,21^. In addition, it was confirmed that the PL-QYs for QDs were the same as the quantum efficiency calculated from the exiton lifetimes for QDs using time-resolved PL decay curves, as shown in Supplementary Fig. S7 (Supporting information)^22^.

**Figure 3:**
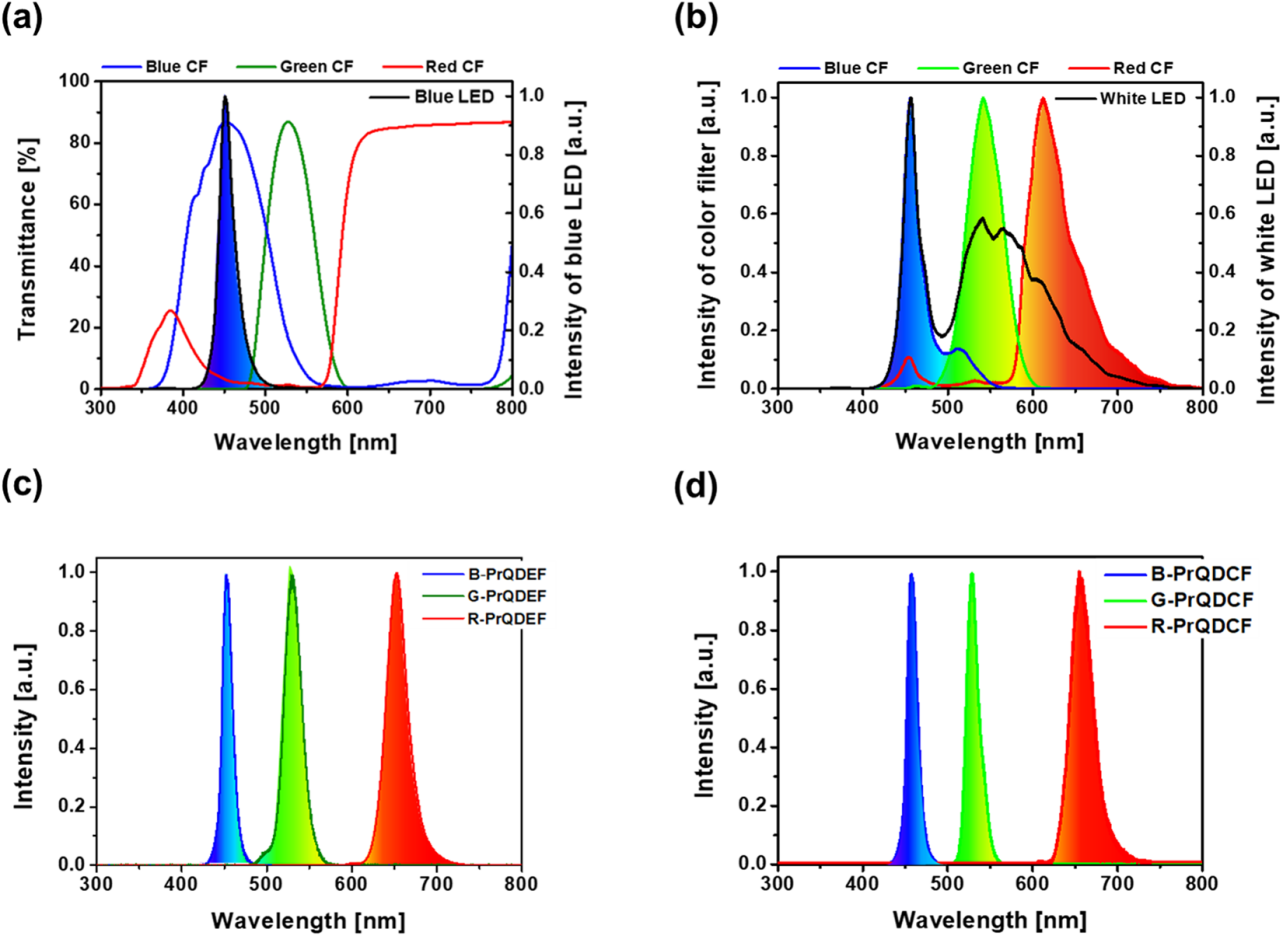
Optical properties of QD functional CF LCD. (**a**) PL spectrum of blue LED and transmittance spectrums of B-CF, G-CF, and R-CF, (**b**) PL spectrums of conventional LCD using conventional B-, G-, and R-CFs and white LED BLU, (**c**) PL spectrums of B-, G-, and R-PrQDEF LCD using blue LED BLU^23^ (**d**) PL spectrums of B-, G-, and R-PrQD functional CF LCD using blue LED BLU.

In order to achieve the best color gamut performance of the QD-functional CF LCD, the emitting light wavelengths from QDs were intentionally and carefully adjusted by the chemical composition and growth parameters in synthesizing QDs since the peaked emitting wavelengths of the B-, G-, and R-PrODs should be exactly tuned with the peaked transmitted wavelengths of B-, G-, and B-CFs. First of all, a blue LED showed the narrow PL spectrums peaked at blue 452-nm in wavelength, which was measured by implementing a blue LED source in the PL tool, as shown in the black solid line in Fig. 3a. Otherwise, B-, G-, and R-functional CFs coated on quartz glass presented the wider transmittance spectrums of 371–563 nm, 478–595 nm, and over 570 nm; and the peaked transmission spectrums of 451 nm, 527 nm, and over 631 nm, respectively.

Second, a conventional LCD using a white LED BLU and conventional R-, G- and B-CFs in Fig. 1a were fabricated by designing proper CF thickness, as shown in Fig. 3b (see Methods part). The conventional LCD showed that the polarized PL peak wavelength and the PL FWHM were 456 nm and 20.6 nm, 542 nm and 49.1 nm, and 612 nm and 58.7 nm for B-, G-, and R-light emission, respectively, as shown in Table 1, resulting in a considerable polarized PL spectrum cross-talk between the B- and G-light emission (i.e., 36 nm) or between the G- and R-light emission (i.e., 73 nm).

**Table 1:** Optical properties, CIE coordinates, and color gamut of conventional LCD, PrQDEF LCD^23^, and PrQD functional CF LCD.

Sample	Peak frequency	PL peak WL [nm]	FWHM [nm]	CIE coordinates [x,y]	Color gamut	
					NTSC [%]	Rec. 2020 [%]
Conventional LCD	Blue	456	20.6	(0.14, 0.10)	73.7	55.1
	Green	542	49.1	(0.29, 0.68)		
	Red	612	58.7	(0.61, 0.33)		
PrQDEF LCD	Blue	452	20.4	(0.15, 0.02)	134.2	100.4
	Green	530	23.8	(0.18, 0.77)		
	Red	652	28.1	(0.73, 0.28)		
PrQD functional CF LCD	Blue	458	14.2	(0.14, 0.03)	137.0	102.7
	Green	528	16.5	(0.17, 0.79)		
	Red	654	29.0	(0.73, 0.28)		

Third, as a comparison, a PrQDEF LCD^23^ in Fig. 1b was fabricated by using the QDEF, i.e. mixing of G- and R PrQDs in a transparent resin) and a blue LED, as shown in Fig. 3c. The PrQDEF LCD presented that the polarized PL peak wavelength and the PL FWHM were 452 nm and 20.4 nm, 530 nm and 23.8 nm, and 652 nm and 28.1 nm for B-, G-, and R-light emission, respectively, as shown in Table 1, resulting in almost free of polarized PL spectrum cross-talk between the B- and G-light emission or between the G- and R-light emission.

Finally, our proposed novel B-, G-, and B-PrQD-functional CF LCD in Fig. 1c was fabricated by using the blended B-, G-, and B-PrQDs in B-, G-, and R-CFs respectively and a blue LED, as shown in Fig. 3d. The B-, G-, and B-PrQDs functional CF LCD exhibited that the polarized PL peak wavelength and the PL FWHM were 458 nm and 14.2 nm, 528 nm and 16.5 nm, and 654 nm and 29.0 nm for B-, G-, and R-light emission, respectively, as shown in Table 1. In particular, the polarized B-, G-, and R-PL peaks (i.e., 458 nm, 528 nm, and 654 nm) in the B-, G-, and B-PrQDs functional CF LCD were red-shift around 10 nm, 17 nm, and 27 nm from the emitting PL peaks (i.e., 448 nm, 511 nm, and 627 nm) for B-, G-, and R-PrQDs in the solvent solution, respectively. This result means that the polarized B-, G-, and R-PL peaks in the B-, G-, and B-PrQD functional CF LCD is red-shifted when the B-, G-, and R-PrQDs in the solvent solution were blended with B-, G-, and R-CFs. In addition, the polarized B-, G-, and R-PL FWHMs (i.e., 14.2 nm, 16.5 nm, and 29.0 nm) for the B-, G-, and B-PrQD functional CF LCD were much narrower than those using the conventional LCD (i.e., 20.6 nm, 49.1 nm, and 58.7 nm) using B-, G-, and B- CF, as shown in Table 1. Note that blue PrQDs in the solution absorbed the light having the wavelength less than ~450 nm so that they emitted the blue light with the FWHM of 20.2 nm at 448-nm in wavelength, called energy-down-shift, as shown Fig. 2d. Otherwise, blue PrQDs in the B-PrQD functional CF only absorbed the light having the wavelength between ~375 and ~450 nm, as shown in the blue shadow region of Supplementary Fig. S8a (Support information). As a result, they emitted the blue light with the FWHM of 14.2 nm at 458-nm in wavelength, which FWHM was narrower than the FWHM of the emitted blue light in the solution (i.e. 20.2 nm), as shown in Supplementary Fig. S8a (Support information). Like a blue PrQDs in the B-PrQD functional CF, green PrQDs in the solution absorbed the light having the wavelength less than ~550 nm so that they emitted the green light with the FWHM of 20.4 nm at 511-nm in wavelength, as shown Fig. 2e. Otherwise, green PrQDs in the G-PrQD functional CF only absorbed the light having the wavelength between ~495 and ~595 nm, as shown in the green shadow region of Supplementary Fig. S8b (Support information). Thus, they emitted the green light with the FWHM of 16.5 nm at 528-nm in wavelength, which FWHM was narrower than the FWHM of the emitted green light in the solution (i.e. 20.4 nm), as shown in Supplementary Fig. S8b (Support information). Both the blue light emitted from the B-PrQD functional CF and the green light emitted from the G-PrQD functional CF showed clearly cross-talk free between the blue and green light, as shown in Fig. 3d. Moreover, the limited light absorption and emission via G-and R-PrQDs in the G-and R-PrQD functional CF from the blue LED BLU greatly improved the cross-talk performance between the green and red light, as shown in Table 1 and Fig. 3d. As a result, the color gamut performance of the B-, G-, and B-PrQD-functional CF LCD would be highly better than the conventional LCD and slightly better than the PrQDEF LCD. Particularly, the polarized PL FWHMs (i.e., 14.2 nm, and 16.5 nm) for the B-and G-PrQD-functional CF LCD were narrower than those for the B-, and G-, PrQDEF LCD (i.e., 20.4 nm and 23.8 nm) while the polarized PL FWHM (i.e., 29.0 nm) for the R-PrQD-functional CF LCD was slightly wider than that for the R-PrQDEF LCD (i.e., 28.1 nm), as shown in Table 1 and Fig. 3c,d. Note that, however, further study is necessary for investigating whether or not the FWHM reduction of B, G, and R-PrQD-Function CFs is related to the QD-size distribution reduction in CFs or composition (i.e., dispersion ability of QDs in R-, G-, and B- CFs or conduction and valance band offset effect between PrQDs and CFs)

**Figure 4:**
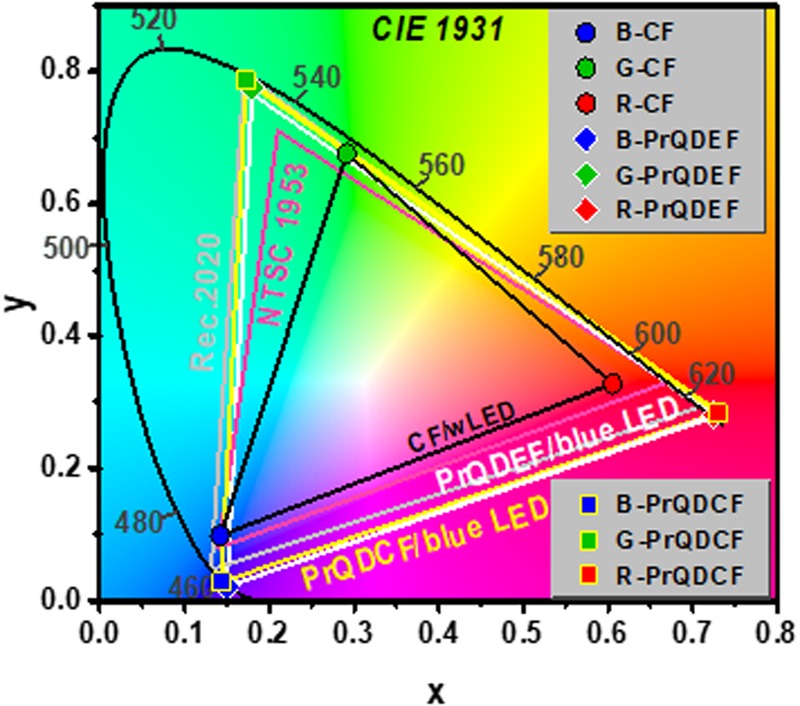
Comparison of the RGB color triangles between conventional LCD under white LCD, PrQDEF LCD, and PrQD functional CF LCD under blue LED in the CIE 1931 color space.

In order to employ this proposed technique for an ultra-high resolution LCD application, the color gamut performance of the PrQD functional CF LCD using a blue LED BLU was estimated by CIE 1931 color space using Rec. 2020 and NTSC 1953 standards^24^, compared with those of the conventional LCD using a white LED BLU and conventional CFs and the PrQDEF LCD using a blue LED BLU, as shown in Fig. 4. The CIE coordinates of three types of LCD were calculated by using the PL spectrum information [Fig. 3b–d] and Matlab-based CIE coordinate calculator software. The RGB color gamut of the conventional LCD using a white LED BLU and conventional R-, G-, and B-CFs is 73.7% (NTSC) and 55.1% (Rec. 2020), which is essentially necessary to be improved for ultra-high resolution LCD application, as shown in Table 1. Otherwise, the RGB color gamut of the PrQD functional CF LCD using a blue LED BLU presented 137.0% (NTSC) and 102.7% (Rec. 2020), which improved remarkably the RGB color gamut compared to the conventional LCD using a white LED and PrQDEF LCD using blue LED. In addition, the RGB color gamut of the PrQDEF LCD using a blue LED BLU presented 134.0% (NTSC) and 104% (Rec. 2020), which was slightly lower than that of the PrQD functional CF LCD using a blue LED BLU (137.0% in NTSC and 102.7% in Rec 2020), as shown in Table 1. Thus, the RGB color gamut of the PrQD functional CF LCD (called the third paradigm shift in the LCD technology) using a blue LED BLU (137.0% in NTSC) demonstrated better than the QDEF LCD (called the second paradigm shift in the LCD technology) using a blue LED BLU and a transparent film mixed with G and R-QDs in a transparent resin (84–103% in NTSC)^25–27^.

## Conclusion

The PrQD functional CF LCD (called the third paradigm shift in the LCD technology) using a blue LED BLU demonstrated the perfect cross-talk-free B-, G-, and R-emitting PL spectrums and extremely wide RGB color gamut. Thus, the PrQD functional CF LCD using a blue LED can overcome a fundamental drawback of the emitting light power loss (>10%) due to using the transparent film including the G- and R-QDs mixed resin and a blue LED BLU in the QDEF LCD (called the second paradigm shift in the LCD technology). In addition, the fabrication process of the PrQD functional CF LCD using a blue LED BLU (i.e., just using B-, G-, and R- PrQD function CFs instead of conventional B-, G-, and R CFs and conventional blue LED) would be simpler than the QDEF LCD adding the transparent QD film mixed with G- and R-QDs and using a blue LED. In particular, the PrQD functional CF would achieve the perfect B-, G-, and R-color gamut (i.e., ~100% in Rec. 2020) when this LCD technology uses CFs well dispersed with R-, G-, and R-PrQDs, the red shifts of the peaked wavelength of the emitting B-, G-, and R-PL spectrum between the B-, G-, and R-PrQD solutions and B-, G-, and R-color filters are properly aligned, and the photolithography technology of the QD functional CFs is developed. Therefore, the QD function CF LCD using a blue LED BLU would lead the third paradigm shift in the ultra-high resolution LCD application, which oversteps the second paradigm shift in the ultra-high resolution LCD application via the QDEF LCD using a blue LED BLU and transparent QD film. In general, the InP based QDs have been widely utilized for the QD film in the QDEF LCD^17^. The InPQD functional CF LCD using G-, and R-InPQD function CFs and a blue LED would perform lower which the RGB color gamut performance than the PrQD functional CF LCD since the FWHM of G- and R-InP QDs are larger than those of G- and R-Pr QDs^28^. However, the B-, G-, and R-PrQDs basically need to improve stability by designing a surfactant during the PrQD synthesis; for an example, PFOA (perfluorooctanoic acid) in our experiment, as shown in Supplementary Figs S9 and S10 (Supporting information). In addition, in a PrQD functional CF LCD, we need to consider the degradation of the ambient contrast ratio by the self-emission of QDs via absorbing the ambient light^25^.

## Electronic supplementary material

Supplementary Information
