# Supplementary material for: Super Ultra-High Resolution Liquid-Crystal-Display Using Perovskite Quantum-Dot Functional Color-Filters
Source: Sci Rep. 2018 Aug 27;8:12881. doi: 10.1038/s41598-018-30742-w (PMC6110735; doi:10.1038/s41598-018-30742-w)
Supplement: Supplementary file 1 — Supplementary Information [file 41598_2018_30742_MOESM1_ESM.doc]

**Super Ultra-High Resolution Liquid-Crystal-Display**

**Using Perovskite Quantum-Dot Functional Color-Filters**

**Yun-Hyuk Ko^1^, Mohammed Jalalah^1^, Seng-Jae Lee^1^ & Jea-Gun Park^*1^**

*^1^Advanced Semiconductor Materials and Devices Center, Department of Electronics and Computer Engineering, Hanyang University,*

*Seoul, 133-791, Republic of Korea.*

*Corresponding author:

Jea-Gun Park

17 Haengdang-dong, Seongdong-gu, Seoul 04763, Republic of Korea

Tel. (+82)-2-2220-0234; fax (+82)-2-2296-1179.

E-mail address: parkjgl@hanyang.ac.kr


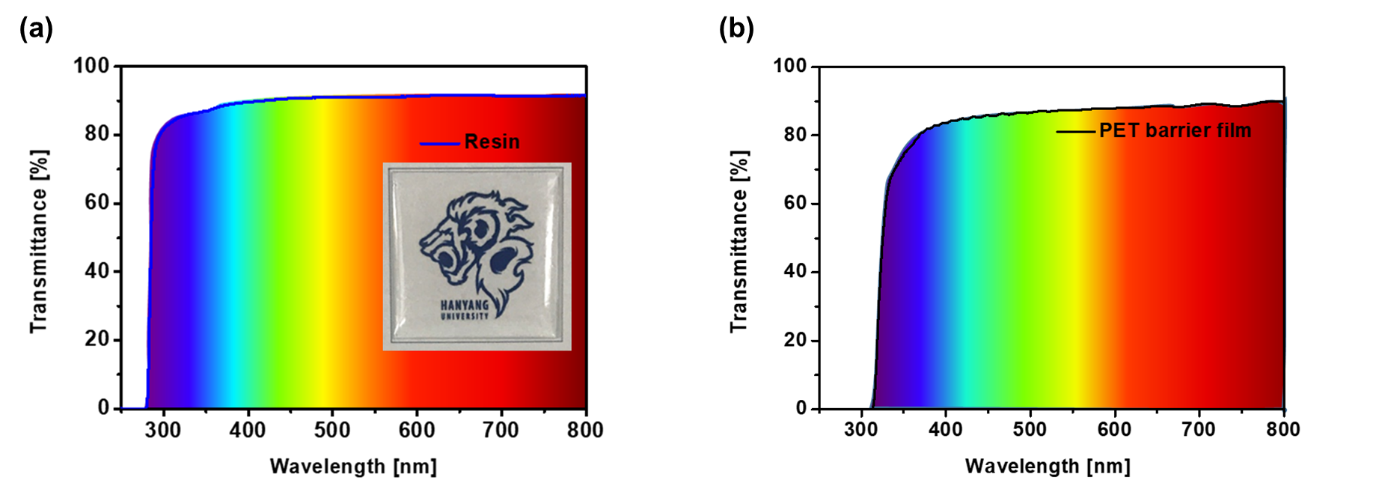


Figure S1. (a) Transmittance spectrum of a resin material coated on a glass substrate. The inset image shows the high transparency of the resin film placed over the Hanyang University logo, (b) Transmittance spectrum of PET barrier film.**^23^**

**
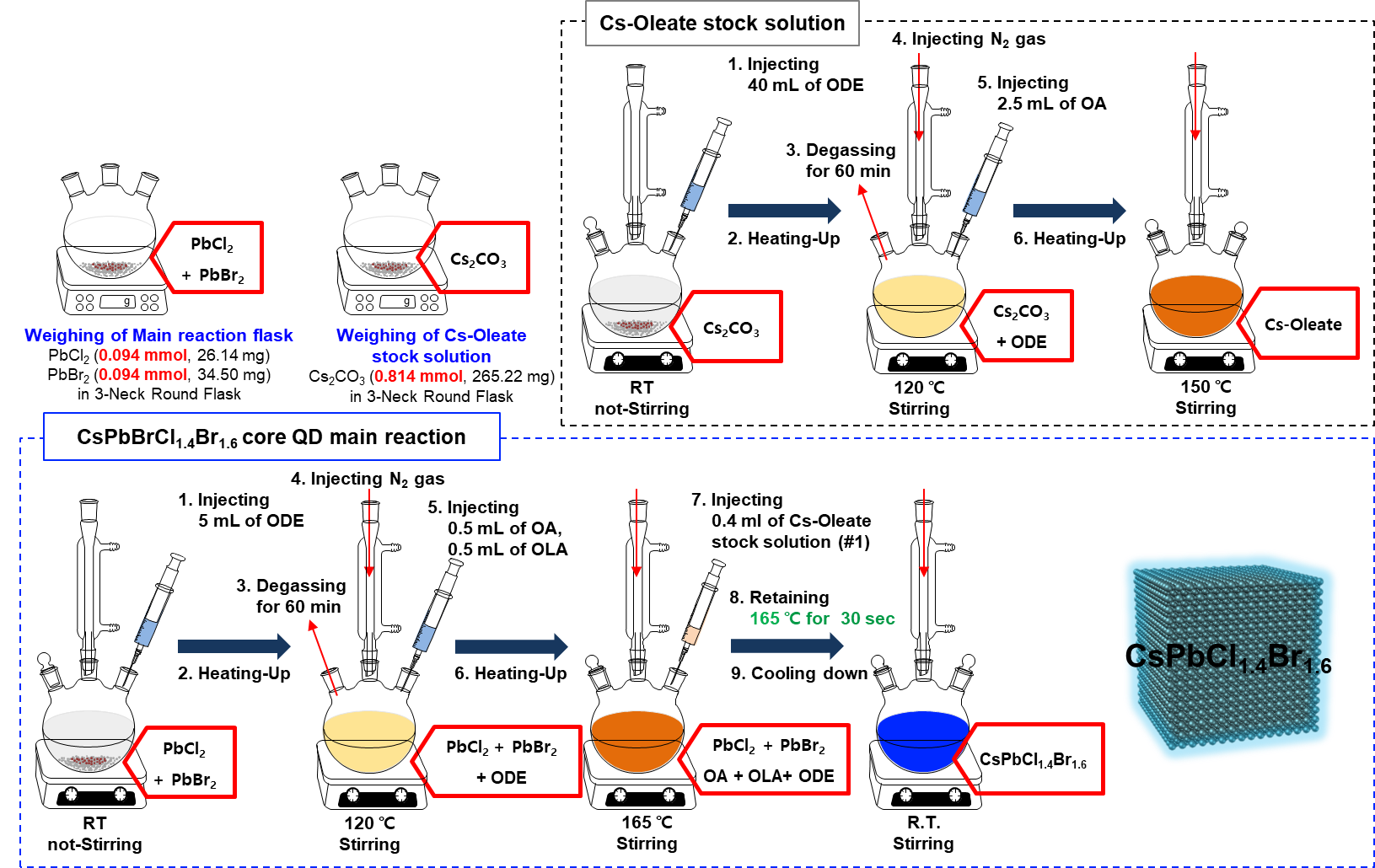
**

Figure S2. Schematic diagram for synthesizing B-PrQD (CsPbCl_1.4_Br_1.6_).

**
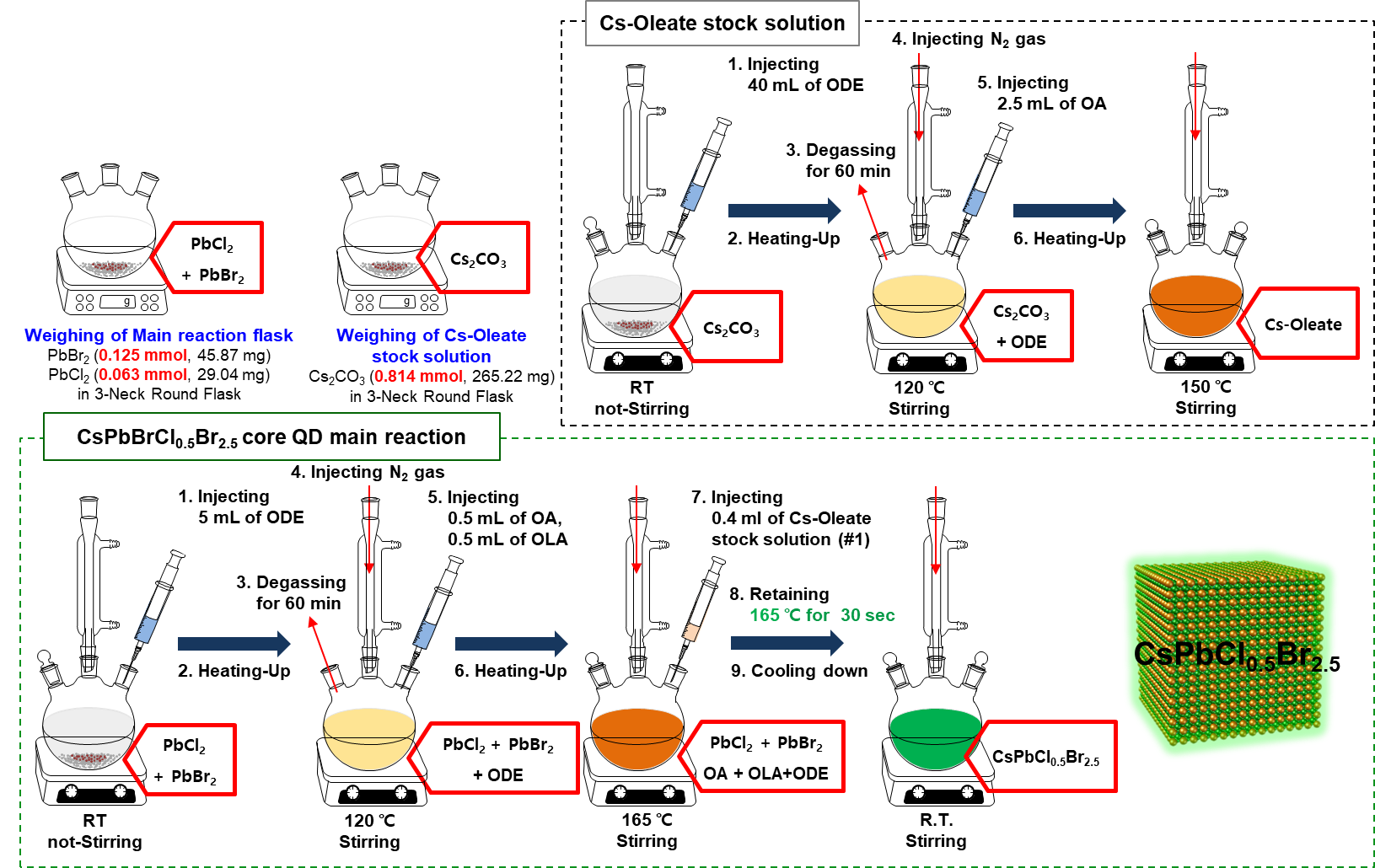
**

Figure S3. Schematic diagram for synthesizing G-PrQD (CsPbCl_0.5_Br_2.5_).

**
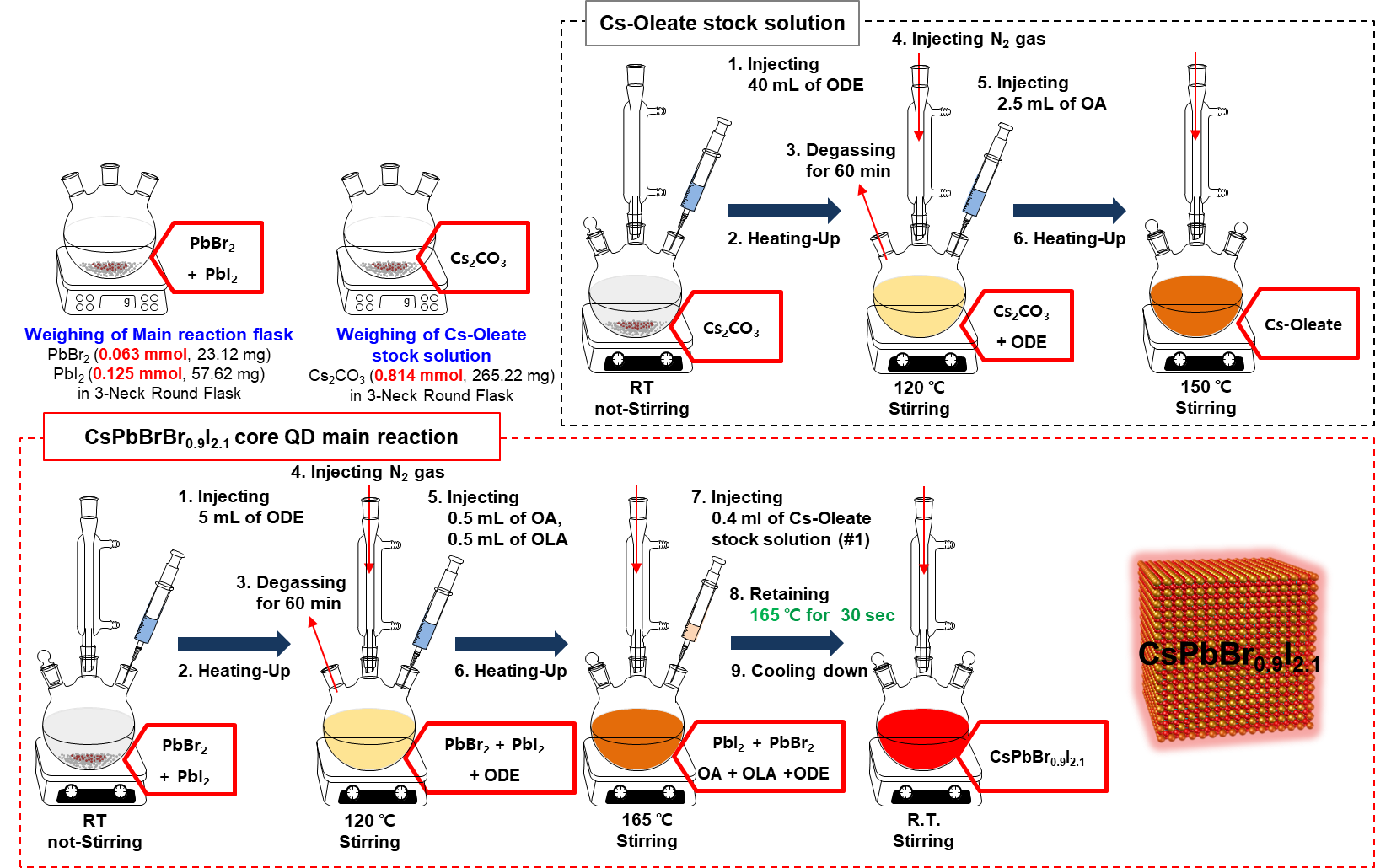
**

Figure S4. Schematic diagram for synthesizing R-PrQD (CsPbBr_0.9_I_2.1_).


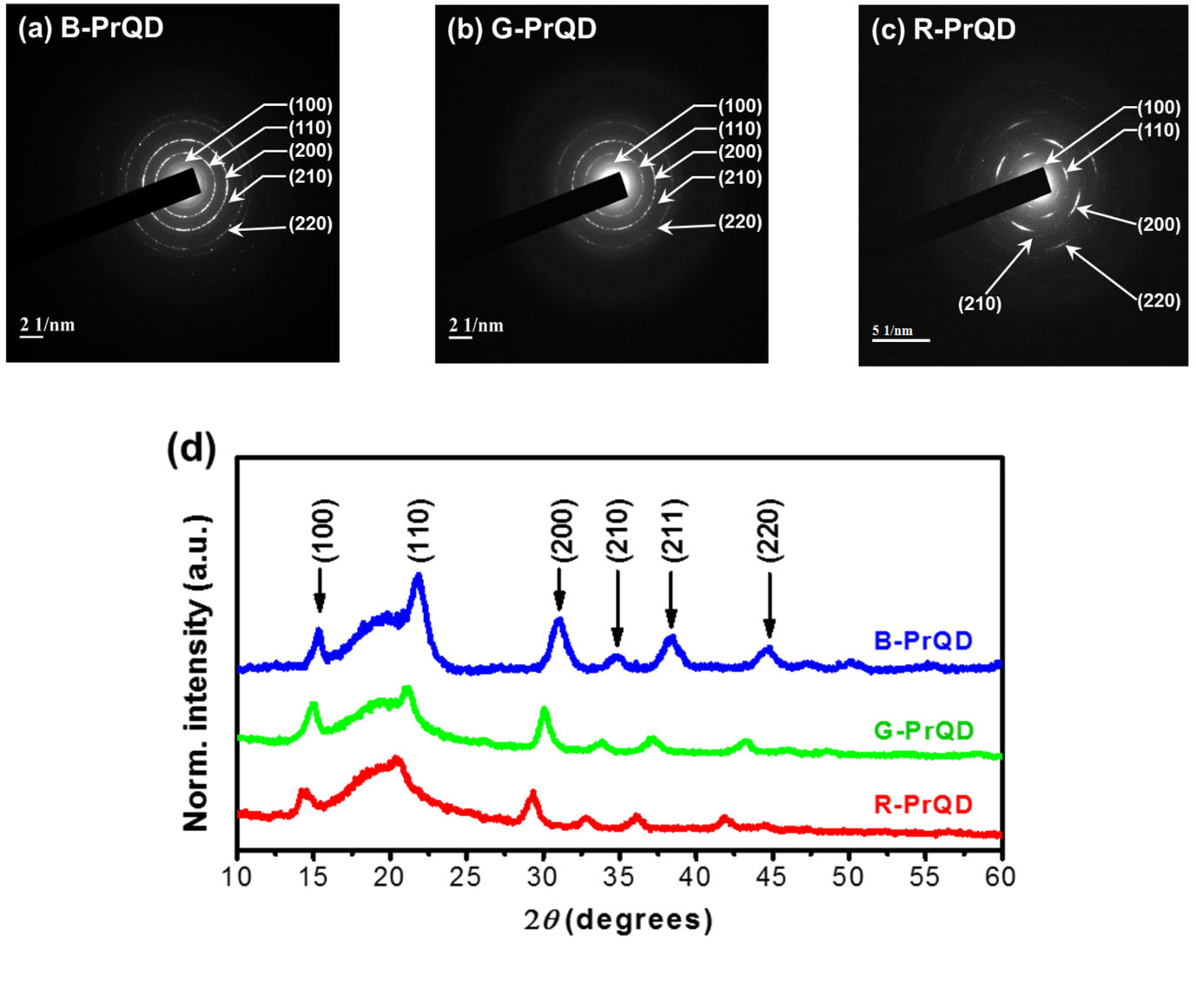
Figure S5. Crystalline properties of perovskite-based QDs. SAED images of (a) B-PrQDs, (b) G-PrQDs, and (c) R-PrQDs; and (d) XRD patterns of B-PrQDs, G- PrQDs, and R-PrQDs in powder form, showing cubic crystalline structure


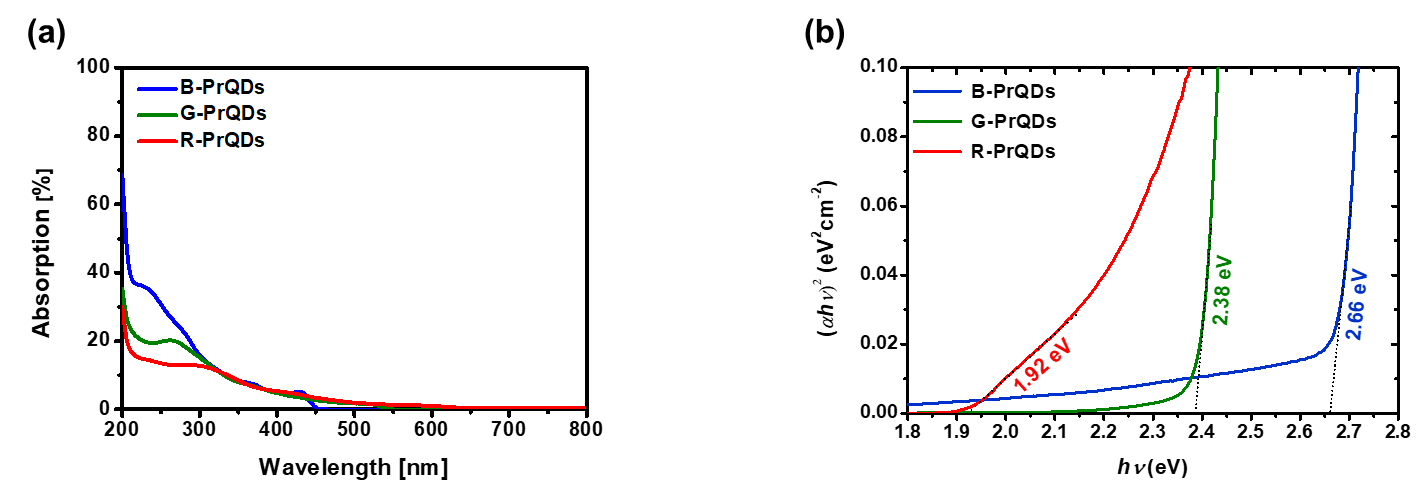


Figure S6. (a) Absorption of B, G, and R-Perovskite QDs correlated with the 10 % absorption for each QDs. (b) Tauc plot of B, G, and R-Perovskite core QDs to calculate their optical band gap. Tauc equation:

*αhν∝(hν-E_g_)^n^* (1)

where *α* is the absorption coefficient, *h* is the Planck constant, *ν* is the frequency, *hν* = 1240/wavelength, and E_g_ is the optical band gap of QDs. Exponent n is a constant and denotes the nature of the transition in the band gap and is 1/2 because of direct band-gap material. The optical band gap of B, G, and R-Perovskite QDs were 1.92, 2.38, and 2.66 eV, respectively.

Figure S7. Time-resolved PL decay curves for the B-, G-, and R-perovskite QDs under 365-nm exitation. The exciton lifetime for B, G, and R-Perovskite QDs were 0.69, 2.12, and 3.39 ns, respectively, as shown in Table 1. Exciton lifetime (τ) from time-correlated single-photon counting (TCSPC) and Quantum efficiency (QE) are given by the equations: **^28^**


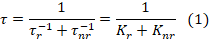


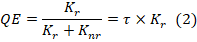


Where *τ_r_*, *τ_nr_*, *K_r_*, and *K_nr_* are radiative recombination time, non-radiative recombination time, radiative rate, and non-radiative rate, respectively. The quantum efficiency of B-, G-, and R-Perovskite QDs were 40.1 %, 61.0 %, and 90.2 %, respectively.

| Samples | B-PrQD | G-PrQD | R-PrQD |
| --- | --- | --- | --- |
| τ (ns) | 0.69 | 2.12 | 3.39 |
| τ_r_ (ns) | 1.85E-2 | 3.48E-2 | 3.76E-2 |
| τ_nr_ (ns) | - | - | - |
| Quantum efficiency (%) | 40.1 | 61.0 | 90.2 |

Table 1. The exciton lifetime, radiative recombination time, non-radiative recombination time, quantum efficiency for B-, G-, and R-perovskite QDs.


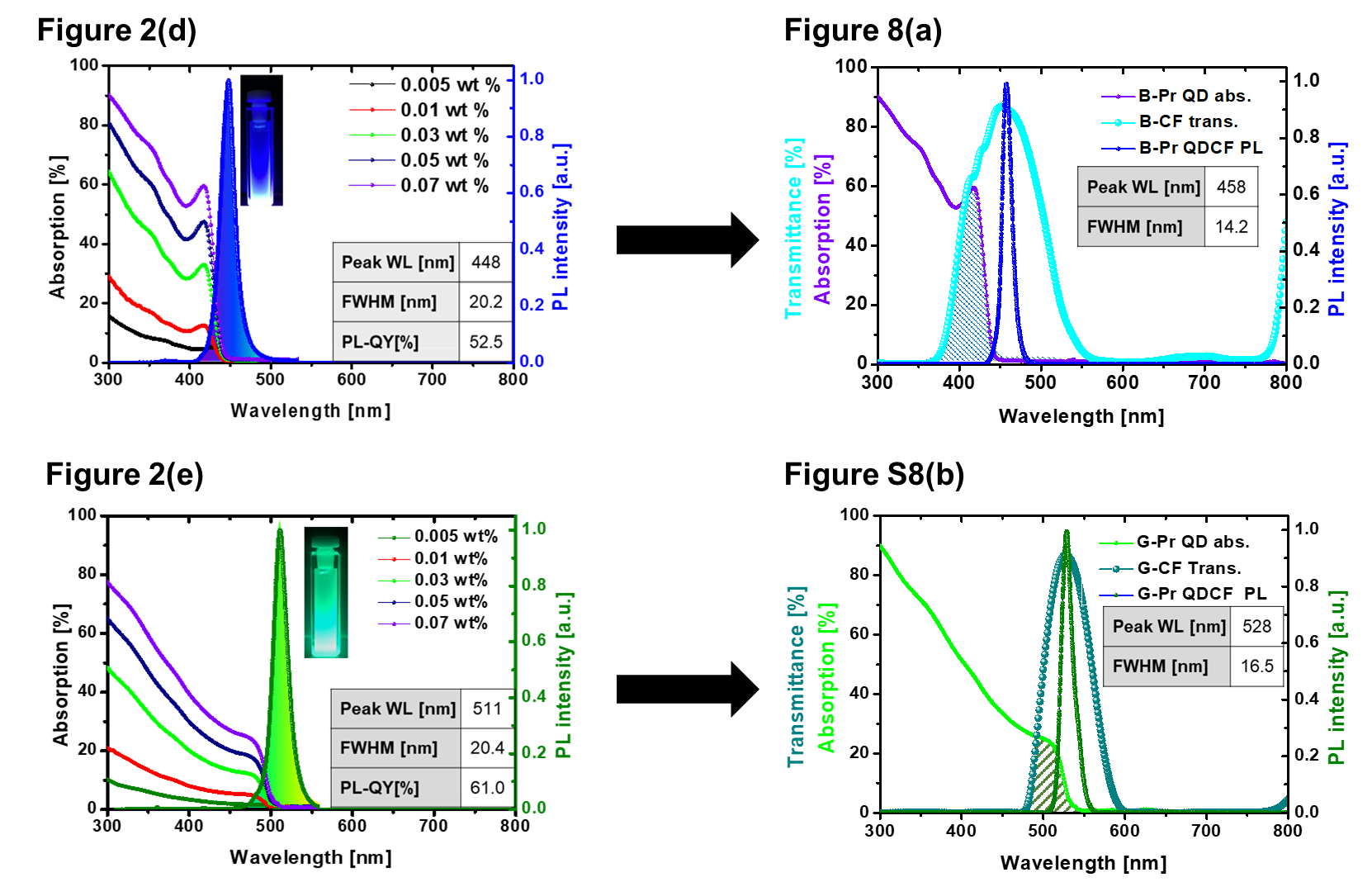


Figure S8. (a). Transmittance spectrum of B-CF(cyan color), absorption of B-PrQD solution(purple color), absorption of B-PrQD functional CF(blue shadow color), and PL of B-PrQD functional CF(blue color).

Figure S8. (b). Transmittance spectrum of G-CF(dark cyan color), absorption of G-PrQD solution(green color), absorption of G-PrQD functional CF(green shadow color), and PL of G-PrQD functional CF(olive color).

Figure 2 (d) and Figure 2 (e) comes from the manuscript

Figure S9. Absolute quantum-yield stability for red CsPbBr_0.9_I_2.1_ PrQD

**
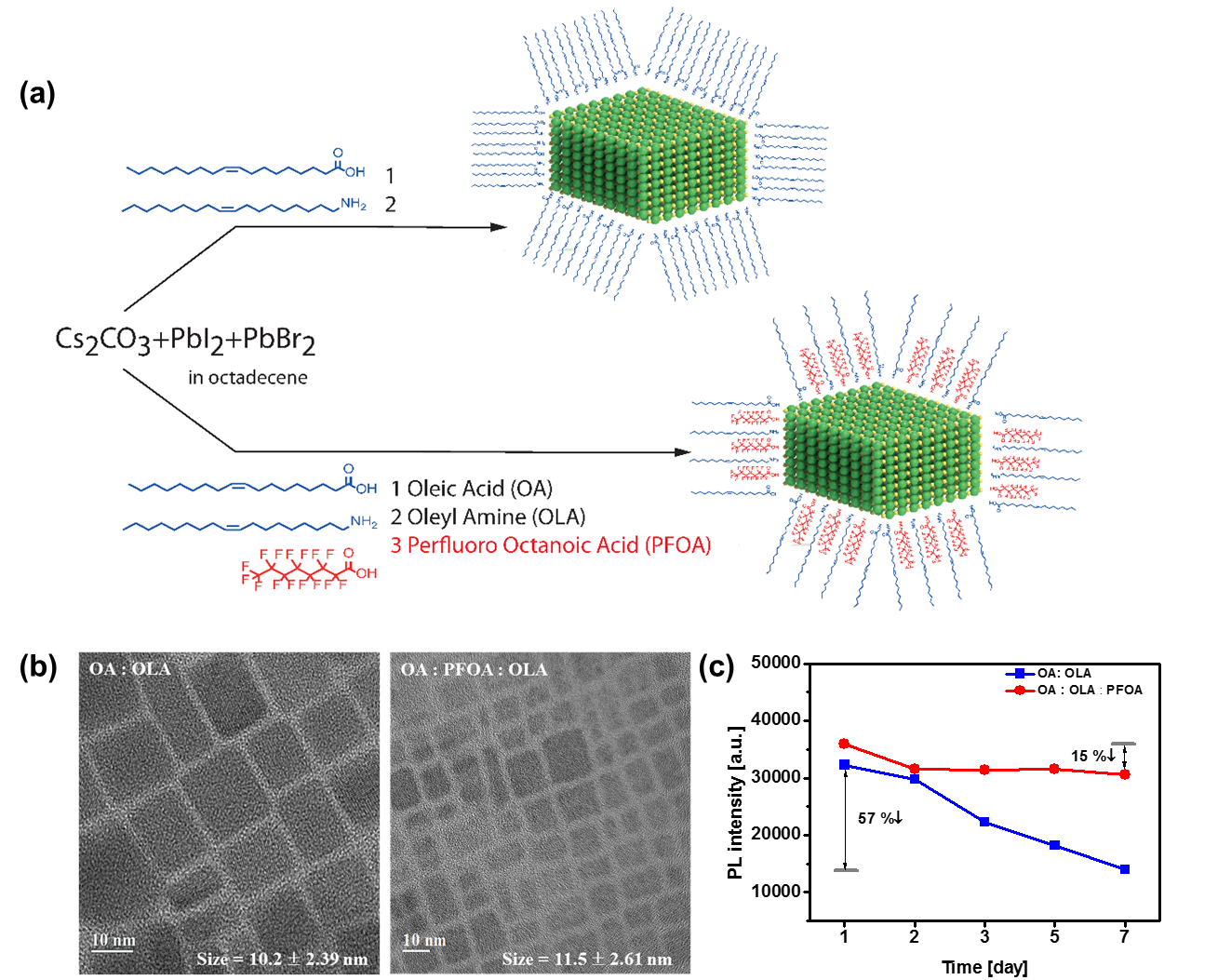
**

Figure S10. (a) Scheme perovskite core QDs with and without PFOA, TEM of perovskite core QDs with (b) and without (c) PFOA, (d) PL intensity depending on the time for perovskite core QDs with and without PFOA.
